# Synergistic Combination of Living Ring-Opening Metathesis Polymerization and Atom Transfer Radical Polymerization to Synthesize Structurally Tailored and Engineered Macromolecular Networks

**DOI:** 10.1021/acs.langmuir.4c03654

**Published:** 2024-12-31

**Authors:** Mohammad Yasir, Brian Hu, Ting-Chih Lin, Krzysztof Matyjaszewski

**Affiliations:** † Department of Chemistry, Carnegie Mellon University, 4400 Avenue, Pittsburgh, Pennsylvania 15213, United States; ‡ Department of Chemistry, Physics, and Atmospheric Sciences, Jackson State University, 1400 Lynch Street, Jackson, Mississippi 39217, United States

## Abstract

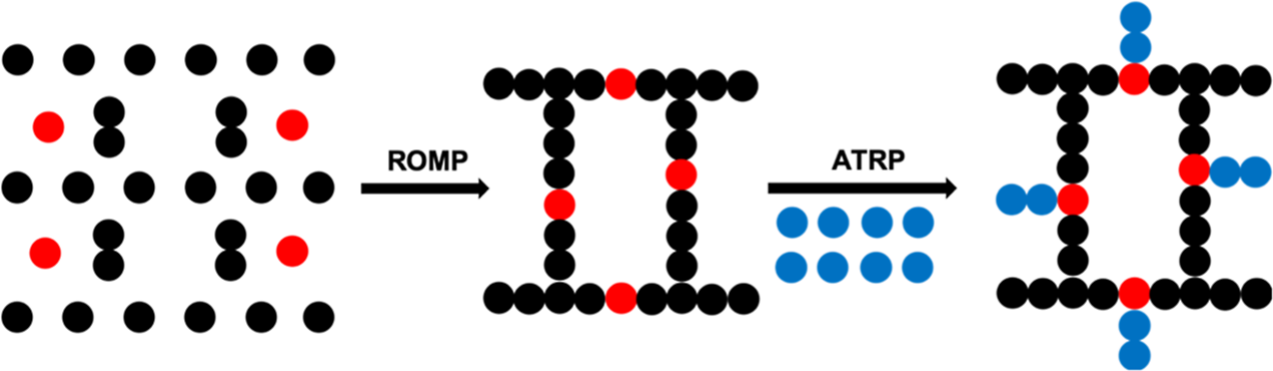

Structurally tailored and engineered macromolecular (STEM) networks are attractive materials for soft robotics, stretchable electronics, tissue engineering, and 3D printing due to their tunable properties. To date, STEM networks have been synthesized by atom transfer radical polymerization (ATRP) or the combination of reversible addition–fragmentation chain-transfer (RAFT) polymerization and ATRP. RAFT polymerization could have limited selectivity with ATRP inimer sites that can participate in radical-transfer processes. On the other hand, living ring-opening metathesis polymerization (ROMP) can produce a polymeric network with latent ATRP initiator sites in high selectivity. Herein, for the first time, we report the syntheses of STEM zero-generation (STEM-0) networks using a monomer, a cross-linker, and an ATRP/ROMP inimer via living ROMP, followed by their modification using a second monomer via ATRP to synthesize STEM first-generation (STEM-1) networks. The mechanical property and swelling capacity analyses of these networks were carried out. A change in mechanical properties and swelling capacity of these networks was observed due to their structural modification.

## Introduction

**Figure 1:**
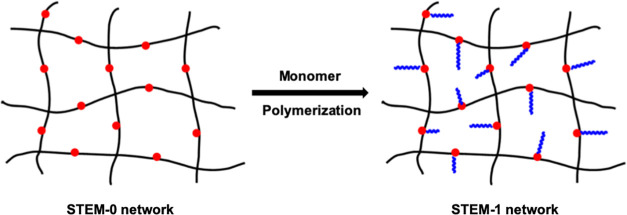
Graphical representation of STEM-0 and STEM-1 networks (red dots: inimer).

Polymer networks containing latent initiator sites available for postsynthesis modification to meet the requirements of multiple applications were termed as structurally tailored and engineered macromolecular (STEM) networks (see Figure 1).^1,2^ The initial network is termed STEM-0, and the modified network, containing polymer side chains, is termed STEM-1. Polymer networks are open systems due to reversible swelling properties, which allow the infiltration of new monomers to form polymeric chains from latent initiator sites.^3^ The initial network is termed STEM-0, while the network after modification containing polymer side chains as STEM-1 networks.

The mechanical properties of STEM networks can be tuned by postsynthesis modification, e.g., a hard polymeric network becomes a soft elastomer after the introduction of low-*T*_g_ grafting polymer side chains.^4−7^ A hydrophobic polymeric network becomes amphiphilic after the introduction of polymer side chains composed of hydrophilic monomers.^3^ Due to these tunable properties, functional networks are attractive materials for tissue engineering,^8,9^ soft robotics,^10,11^ stretchable electronics,^12^ and 3D printing.^13^

STEM networks can be synthesized by free radical polymerization (FRP). However, in FRP, the formation of high molecular weight fractions take place more rapidly and heterogeneously, thus giving poor control over mesh size (i.e., network segments or distance between cross-link points) and the architecture of the network.^1^ Controlled radical polymerization (CRP) allows for the synthesis of STEM networks with a more controlled mesh size and homogeneous network architecture than that prepared by FRP.^14−19^ Additionally, CRP processes can be controlled by external stimuli, such as light, affording spatiotemporal control and simplifying reaction setup.^20−25^ Currently, the syntheses of STEM-0 and STEM-1 networks are achieved by reversible addition–fragmentation chain-transfer (RAFT) polymerization and atom transfer radical polymerization (ATRP), respectively.^3^ The combination of both CRP techniques allows the direct incorporation of ATRP inimer in the network, thus avoiding the postsynthesis deprotection step.^3^ RAFT polymerization could have limited selectivity with ATRP inimer sites that can participate in radical-transfer processes. On the other hand, living ring-opening metathesis polymerization (ROMP) can produce a polymeric network with latent ATRP initiator sites in high selectivity.^26^ Therefore, a synthetic route based on living ROMP and ATRP to synthesize STEM-0 and STEM-1 networks should be very efficient. Additionally, living ROMP has faster polymerization kinetics, particularly in the case of strained cyclic olefins.^27−31^ Moreover, living ROMP can be carried out at room temperature and has high functional group tolerance.^32−38^

Here, for the first time, we report the synthesis of a STEM-0 network via living ROMP, followed by its modification via photoinduced ATRP to synthesize STEM-1 networks. The gravimetric swelling capacity and mechanical property analyses of these networks were performed. The mechanical properties and swelling capacity of these networks were modified due to their structural changes.

## Results and Discussion

**Figure 2:**
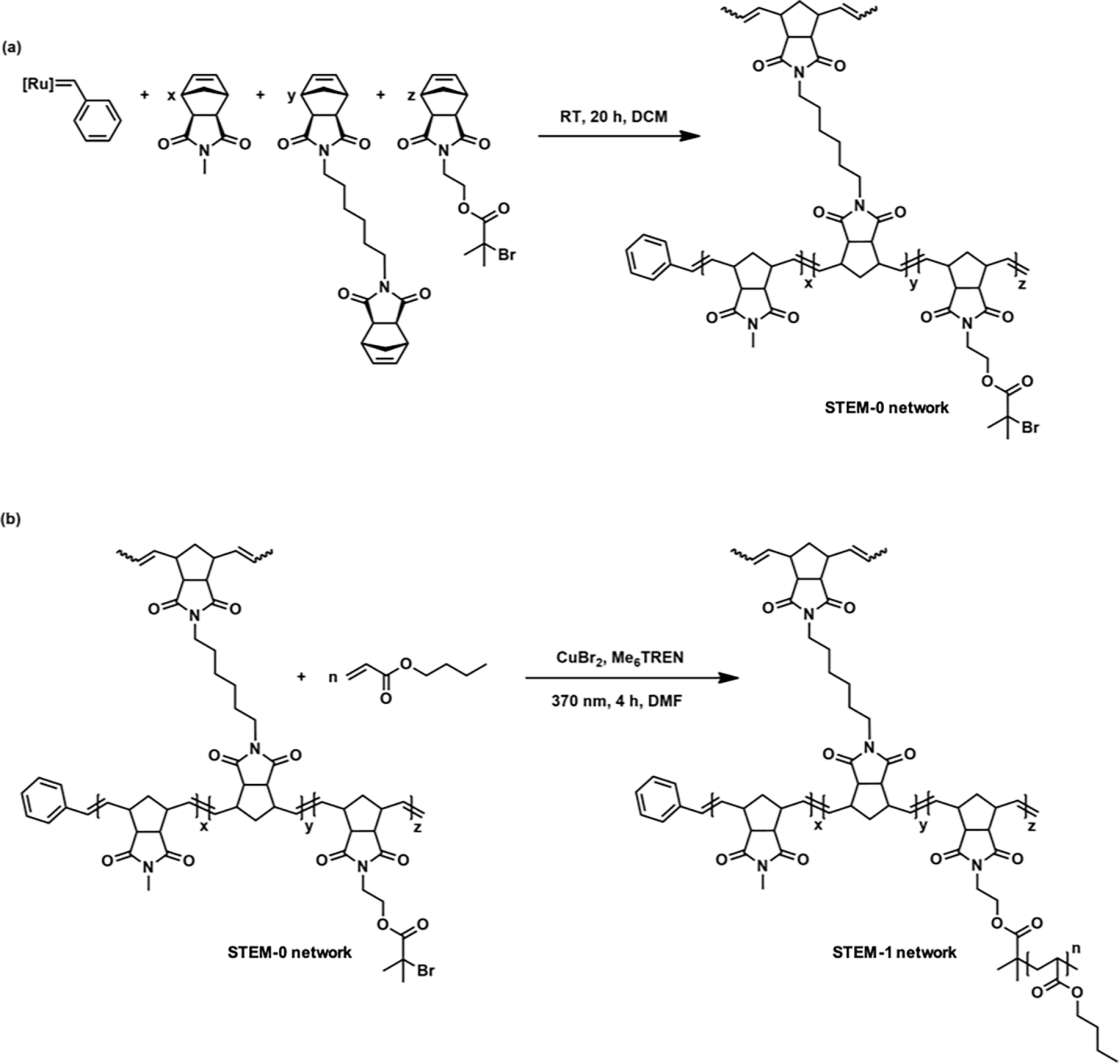
Synthesis of (a) STEM-0 networks via living ROMP and (b) STEM-1 networks via ATRP (see the Supporting Information). (*x* = 450, *y* = 3, and *z* = 50: STEM-0-I10), (*x* = 450, *y* = 3, and *z* = 50, *n* = 100: STEM-1-I10), (*x* = 375, *y* = 3, and *z* = 125: STEM-0-I25), and (*x* = 375, *y* = 3, *z* = 125, and *n* = 100: STEM-1-I25). The detailed structure of the Ru catalyst (G1) is shown in the Supporting Information (see Figure S1).

First, we synthesized the monomer (*exo*-*N*-methyl-norbornenecarboximide), inimer (*exo*-*N*-ethyl-norbornenecarboximide α-bromoisobutyrate), and cross-linker (hexamethylene-*bis*-*exo*-norbornenecarboximide) (cf. Figure 2 and see the Supporting Information).

Next, we employed the Ru-based Grubbs’ first-generation catalyst/initiator (G1) (5 mg, 0.006 mmol, 1 equiv) to react with the monomer (450 equiv), cross-linker (3 equiv), and inimer (50 equiv) under an inert (N_2_) atmosphere in dry dichloromethane (DCM) to produce the STEM-0-I10 network with a theoretical mesh size of 167 (monomer and inimer-to-cross-linker molar ratio) and 10 mol % inimer content (see Figure 2a). This reaction followed typical living ROMP. The sol part of the network was removed by extraction with chloroform, and the network was dried. The gel fraction of STEM-0-I10 was >99% as calculated by the given formula, gel fraction (%) = (*W*_d_/*W*_t_)×100, where *W*_t_ and *W*_d_ are the weights of the dried network before and after removing sol, respectively.

Subsequently, the STEM-0-I10 network (134 mg, containing 24 mg inimer sites, 0.0674 mmol, 1 equiv) was reacted with *n*-butyl acrylate (secondary monomer (100 equiv)) in the presence of CuBr_2_ (0.09 equiv), *tris*[2-(dimethylamino)ethyl]amine (Me_6_TREN) (0.54 equiv), and UV light (370 nm) under an inert (N_2_) atmosphere in degassed *N*,*N*-dimethylformamide (DMF) to synthesize the STEM-1-I10 network with poly(*n*-butyl acrylate) (PBA) side chains (see Figure 2b) using ATRP. The STEM-1-I10 network was washed with chloroform and dried. The ^1^H NMR spectra revealed 56% conversion of *n*-butyl acrylate, resulting in a PBA side chain length of 56 in STEM-1-I10.

We investigated the change of mechanical properties and swelling capacities of STEM-0-I10 versus postmodification STEM-1-I10 networks. The STEM-0-I10 and STEM-1-I10 networks were subjected to compression testing for determination of the mechanical properties. The sample was first loaded with 10 N force, which then linearly increased to 20 N. Stress was calculated with eq 1, where σ is stress in pascals, F is measured force, and A is the area of the sample in contact with the measuring geometry.
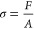
1

Strain was calculated by dividing the gap size at a given force by the initial gap size, which was extrapolated linearly to 0 N.

**Figure 3:**
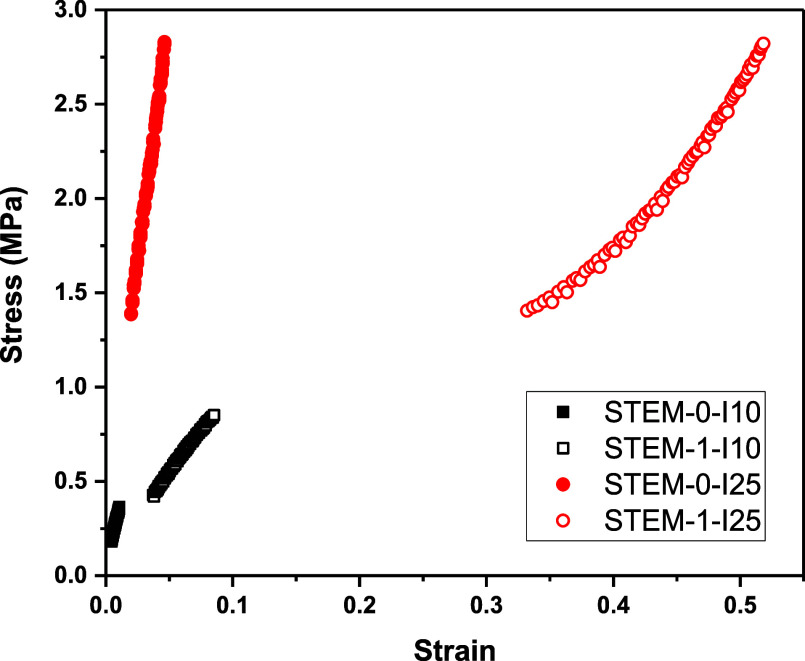
Stress vs strain plot of STEM networks.

The STEM-0-I10 network showed a Young’s modulus value of 28 MPa (see Figure 3, black, closed), while the Young’s modulus value for the STEM-1-I10 network decreased to 9 MPa (see Figure 3, black, open). The Young’s modulus for the STEM-1-I10 network was approximately 3 times lower than that for the STEM-0-I10 network. Thus, the STEM-1-I10 network became softer than the STEM-0-I10 network after the incorporation of low *T*_g_ PBA side chains.

**Figure 4:**
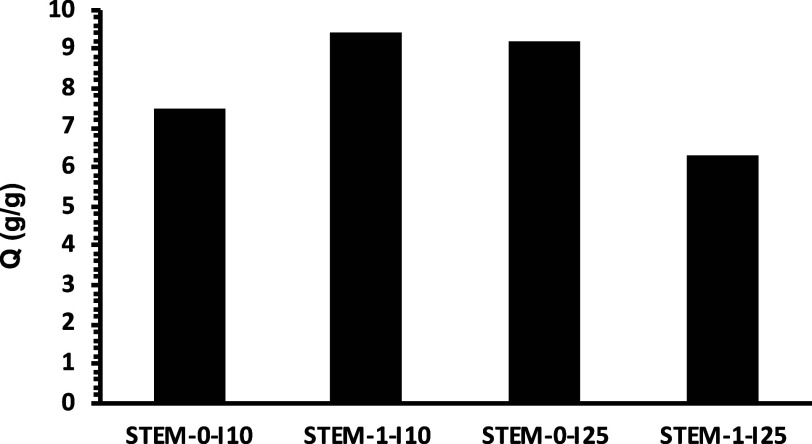
Plot showing swelling capacities of STEM networks (see Table S1, Supporting Information).

To calculate the gravimetric swelling capacities, STEM-0-I10 and STEM-1-I10 networks were subjected to swelling in DMF. The swelling capacity for the STEM-0-I10 network was 7.5, while for the STEM-1-I10 network, it was 9.4 (see Figure 4). We recently showed that a given network has different swelling capacities in different solvents, indicating different affinity of its structure toward different solvents.^38^ Even though the STEM-1-I10 network has less mesh size because of the presence of PBA compared with the STEM-0-I10 network, this increase in swelling capacity could be attributed to higher affinity of the STEM-1-I10 network structure toward DMF than the STEM-0-I10 network structure.

Next, the STEM-0-I25 network with the same mesh size, i.e., 167, but higher inimer content, i.e., 25%, was synthesized. Then, it was modified with *n*-butyl acrylate (23% conversion, see the Supporting Information) to yield the STEM-1-I25 network having a PBA side chain length of 23 (see the Supporting Information). The STEM-0-I25 network exhibited a Young’s modulus of 52 MPa (see Figure 3, red, closed), while the Young’s modulus for the STEM-1-I25 network was determined to be 5 MPa (see Figure 3, red, open). The Young’s modulus for the STEM-1-I25 network was almost 10 times smaller than the STEM-0-I25 network. Thus, the STEM-1-I25 network was softer than the STEM-0-I25 network after incorporating low *T*_g_ PBA side chains. The swelling capacity value in DMF solvent for the STEM-0-I25 network was 9.2, while for the STEM-1-I25 network, it was 6.3 (see Figure 4). This decrease in swelling capacity value is due to the reduced mesh size of the STEM-1-I25 network compared with the STEM-0-I25 network.

## Conclusions

The monomer, inimer, and cross-linker were synthesized and subsequently polymerized via living ROMP to produce STEM-0-I10 and STEM-0-I25 networks. The STEM-0-I10 and STEM-0-I25 networks were modified via ATRP using a secondary monomer (*n*-butyl acrylate) to synthesize STEM-1-I10 and STEM-1-I25 networks. The mechanical property and swelling capacity analyses of these networks were carried out. The Young’s modulus of STEM-1-I10 and STEM-1-I25 networks were approximately 3 and 10 times lower than STEM-0-I10 and STEM-0-I25 networks (9 vs 28 MPa and 5 vs 52 MPa), respectively, due to the incorporation of soft poly(*n*-butyl acrylate) side chains in STEM-1 networks. The swelling capacity of the STEM-1-I10 network was higher than that of the STEM-0-I10 network, possibly due to the greater affinity of the STEM-1-I10 network structure toward DMF solvent than the STEM-0-I10 network structure. The swelling capacity of the STEM-1-I25 network was lower than that of the STEM-0-I25 network due to the reduced mesh size of the STEM-1-I25 network compared with the STEM-0-I25 network.

## Supporting Information Available

Materials, instrumentation, experimental procedures, and NMR Data (PDF)
