# Supplementary material for: Synergistic Combination of Living Ring-Opening Metathesis Polymerization and Atom Transfer Radical Polymerization to Synthesize Structurally Tailored and Engineered Macromolecular Networks
Source: Langmuir. 2024 Dec 31;41(1):378–82. doi: 10.1021/acs.langmuir.4c03654 (PMC11736835; doi:10.1021/acs.langmuir.4c03654)
Supplement: Supplementary file 1 — la4c03654_si_001.pdf [file la4c03654_si_001.pdf]

Supporting Information

Synergistic combination of living ring-opening metathesis polymerization and atom transfer radical polymerization to synthesize structurally tailored and engineered macromolecular networks

Mohammad Yasir,[a,b,*] Brian Hu,[a] Ting-Chih Lin,[a] and Krzysztof Matyjaszewski[a,*]

[a]Department of Chemistry, Carnegie Mellon University, 4400 Avenue, Pittsburgh, PA 15213, USA
[b]Department of Chemistry, Physics, and Atmospheric Sciences, Jackson State University, 1400 Lynch Street, Jackson, MS 39217, USA

*Correspondence: mohammad.yasir@jsums.edu, km3b@andrew.cmu.edu

Table of Contents

Materials **2**

Instrumentation **2**

Experimental procedures **2**

Synthesis of monomer (*exo*-*N*-methyl-norbornenecarboximide): **2**

Synthesis of exo-*N*-hydroxyethyl-norbornenecarboximide: **3**

Synthesis of inimer (*exo*-*N*-ethyl-norbornenecarboximide *α*-bromoisobutyrate): **3**

Synthesis of crosslinker (hexamethylenebis-*exo*-norbornenecarboximide): **3**

Synthesis of STEM-0-I10 network: **4**

Synthesis of STEM-1-I10 network: **4**

Synthesis of STEM-0-I25 network: **4**

Synthesis of STEM-1-I25 network: **4**

General procedure for the swelling capacities analysis: **5**

NMR Data **6**

Materials

Grubbs' first generation catalyst (G1) (Sigma Aldrich, 97%), *cis*-norbornene-*exo*-2,3-dicarboxylic anhydride (Biosynth), methylamine solution, 40 wt. % in H_2O (Sigma Aldrich), ethanolamine (Sigma Aldrich, ≥98%), triethylamine (Sigma Aldrich, ≥99.5%), *α*-bromoisobutyryl bromide (Sigma Aldrich, 98%), hexamethylenediamine (Sigma Aldrich, 98%), copper(II) bromide ($CuBr_2$) (Sigma Aldrich, 99%), tris[2-(dimethylamino)ethyl]amine (Me_6TREN) (Sigma Aldrich, 97%), *n*-butyl acrylate (Sigma Aldrich, ≥98%), *N*,*N*-dimethylformamide (DMF) (Sigma Aldrich), toluene (Sigma Aldrich), dichloromethane (Sigma Aldrich) and deuterated chloroform ($CDCl_3$) (Cambridge Isotope Laboratories, 99.8%) were used as received.

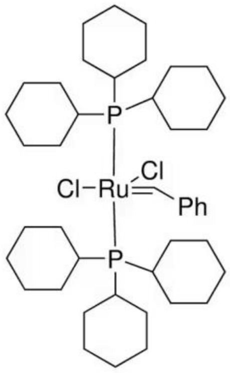

Figure S1. Structure of G1.

Instrumentation

NMR spectra were recorded on a Bruker Avance III NMR spectrometer (^{1}H NMR: 500 MHz; ^{13}C NMR: 101 MHz). Mechanical properties of the polymer networks were assessed in the dry state using an Anton Paar MCR-302 Rheometer (Anton Paar, Graz, Austria) fitted with a 25 mm diameter stainless-steel parallel plate tool. The networks were characterized using disk-shaped samples with a thickness of 2.30 (STEM-1 gel)-4.11 (STEM-0 gel) mm and a diameter of 5.45 (STEM-1 gel)-8.35 (STEM-0 gel) mm.

Experimental procedures

Synthesis of monomer (*exo*-*N*-methyl-norbornenecarboximide):

Cis-norbornene-*exo*-2,3-dicarboxylic anhydride (10 g, 0.0609 mol, 1 eq) was taken in a high-pressure tube having a stirring magnet. Then, 100 mL of toluene was added to the tube, and the mixture was heated at 80 °C with stirring for 10 min to dissolve the anhydride. Finally, methylamine (40-weight % solution in water, 7.88 mL, 1.5 eq) was added to the tube and sealed. The reaction mixture was heated at 110 °C with stirring overnight (ON). The reaction mixture was cooled to room temperature and concentrated under reduced pressure using rotary evaporation, and the resulting solid was dissolved in ethyl acetate. The solution was washed with 0.1 M HCl (2x30 mL), distilled water (2x20 mL), and brine (20 mL). The solution was dried with $MgSO_4$ and concentrated under reduced pressure using rotary evaporation. The resulting solid was recrystallized in the solution of ethyl acetate (20 mL) and hexane (30 mL) to give the product as white solid (76 % yield).

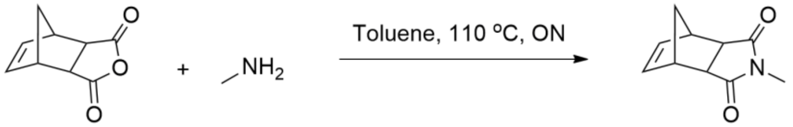

Synthesis of exo-*N*-hydroxyethyl-norbornenecarboximide:

Cis-norbornene-*exo*-2,3-dicarboxylic anhydride (15 g, 0.0914 mol, 1 eq) was taken in a high-pressure tube having a stirring magnet. Then, 150 mL of toluene was added to the tube, and the mixture was heated at 80 °C, stirring for 10 minutes to dissolve the anhydride. Finally, ethanolamine (8.31 mL, 1.5 eq) and triethylamine (1.28 mL, 0.2 eq) were added to the tube and sealed. The reaction mixture was heated at 130 °C with stirring overnight. The reaction mixture was cooled to room temperature and concentrated under reduced pressure using rotary evaporation and the resulting solid was dissolved in dichloromethane (300 mL). The solution was washed with 0.1 M HCl (2x30 mL), distilled water (2x30 mL), and brine (30 mL). The washed solution was added in excess hexane to form the precipitate. The precipitate was filtered by filter paper and dried overnight at room temperature to give the product a white solid (70% yield).

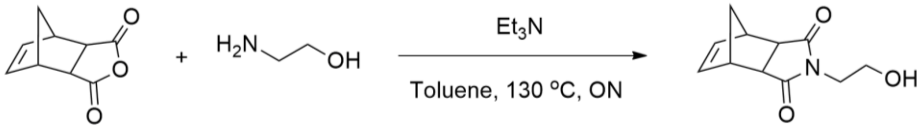

Synthesis of inimer (*exo*-*N*-ethyl-norbornenecarboximide *α*-bromoisobutyrate):

exo-*N*-hydroxyethyl-norbornenecarboximide (13.33 g, 0.0643 mol, 1 eq), triethylamine (9.87 mL, 1.1 eq), and dichloromethane (DCM) (200 mL) were added in a flask having stirring magnet. The flask was sealed by a septum using tape. The tube was made free from the air by purging N_2 through it for 15 min. Then, the flask was cooled in an ice-water bath, and α-bromoisobutyryl bromide (8.27 mL, 1.05 eq) was added dropwise using a syringe in the flask. The reaction mixture was stirred for 48 hours at room temperature (RT). The residue was filtered by filter paper, and the filtrate was washed with the following (30 mL for each wash): two times with 0.1 M HCl (2x30 mL), saturated NaHCO3 solution (4x30 mL), and distilled water (2x30 mL). The solution was dried with $MgSO_4$ and concentrated under reduced pressure using rotary evaporation. The resulting solid was recrystallized in methanol to give the product as white solid (63% yield).

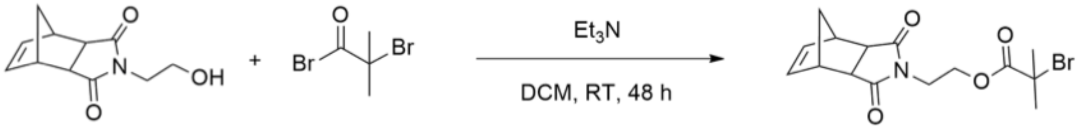

Synthesis of crosslinker (hexamethylenebis-*exo*-norbornenecarboximide):

Cis-norbornene-*exo*-2,3-dicarboxylic anhydride (2 g, 2.5 eq), hexamethylenediamine (0.57 g, 0.0049 mol, 1 eq) triethylamine (0.05 mL, 0.2 eq) and toluene (25 mL) were added in a high-pressure tube having stirring magnet and the tube was sealed. The reaction mixture was heated at 130 °C with stirring overnight. The reaction mixture was cooled to room temperature and concentrated under reduced pressure using rotary evaporation. The resulting solid was purified by column chromatography (eluent: ethyl acetate/hexane=30/70) to give the product as a white solid (33% yield).

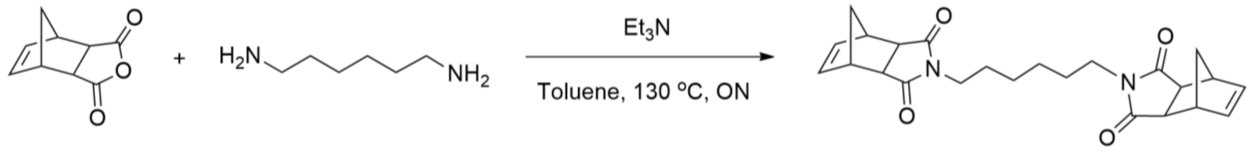

Synthesis of STEM-0-I10 network:

G1 (5 mg, 0.006 mmol, 1 eq) was taken in a 7 mL vial, and the vial was sealed with a septum. The vial was degassed by purging N_2 through it for 10 min. Then, the G1 was dissolved in 0.5 mL of dry DCM. In a separate vial, monomer (450 eq), crosslinker (3 eq), and inimer (50 eq) were taken and dissolved in 1.5 mL of dry DCM. This solution was transferred to the vial containing G1 solution. The reaction mixture was left at room temperature for 20 h. The vial was broken, and the STEM-0-I10 gel was taken out. The network was washed with chloroform (3x20 mL) and left for drying in a vial capped with aluminum foil with holes overnight. Then it was dried in the desiccator overnight. The conversion was found to be quantitative, as the negligible residue was left after the evaporation of the solvent used for washing.

Synthesis of STEM-1-I10 network:

The 134 mg of STEM-0-I10 network (24 mg inimer sites, 1 eq) were taken in a 20 mL vial, and the vial was sealed with a septum. The vial was degassed by purging N_2 through it for 10 min. In a separate 7 mL vial, $CuBr_2$ (0.09 eq), Me_6TREN (0.54 eq), and *n*-butyl acrylate (secondary monomer) (100 eq) were taken and dissolved in 2.6 mL of DMF. The vial was sealed with a septum and degassed by purging N_2 through it for 10 min. This solution was transferred to the STEM-0-I10 gel-containing vial via syringe. The gel was left for infiltration overnight. The gel absorbed a part of the solution. The extra solution was taken out via a syringe. The vial was exposed to UV light (370 nm) for 4 h. The vial was broken, and the STEM-1-I10 gel was transferred to a petri dish. The 2 mL of $CDCl_3$ were added to the petri dish and left for 10 min to dissolve unreacted monomer and DMF to calculate the conversion. The $CDCl_3$ was transferred to the NMR tube. The network was washed with chloroform (3x20 mL) and left for drying in a beaker capped with aluminum foil with holes overnight. Then it was dried in the desiccator overnight. The ^{1}H NMR spectra (before and after the reaction) revealed 56% conversion (DMF acted as the NMR standard).

Synthesis of STEM-0-I25 network:

G1 (5 mg, 0.006 mmol, 1 eq) was taken in a 7 mL vial and sealed with septum. The vial was degassed by purging N_2 through it for 10 min. Then, the G1 was dissolved in 0.5 mL of dry DCM. In a separate vial, monomer (375 eq), crosslinker (3 eq), and inimer (125 eq) were taken and dissolved in 1.5 mL of dry DCM. This solution was transferred to the vial containing G1 solution. The reaction mixture was left at room temperature for 20 h. The vial was broken, and the STEM-0-I10 gel was taken out. The network was washed with chloroform (3x20 mL) and left for drying in a vial capped with aluminum foil with holes overnight. Then it was dried in the desiccator overnight. The conversion was quantitative, as the negligible residue was left after the evaporation of the solvent used for washing.

Synthesis of STEM-1-I25 network:

The 219 mg of STEM-0-I25 network (50 mg inimer sites, 1 eq) were taken in a 20 mL vial, and the vial was sealed with a septum. The vial was degassed by purging N_2 through it for 10 min. In

a separate 7 mL vial, $CuBr_2$ (0.09 eq), Me_6TREN (0.54 eq), and butyl acrylate (secondary monomer) (100 eq) were taken and dissolved in 4.3 mL of DMF. The vial was sealed with a septum and degassed by purging N_2 for 10 min. This solution was transferred to the STEM-0-I25 gel-containing vial via a syringe. The network was left for infiltration overnight. The material absorbed a part of the solution. The extra solution was taken out via a syringe. The vial was exposed to UV light (370 nm) for 4 h. The vial was broken, and the STEM-1-I25 network was transferred to a petri dish. The 2 mL of $CDCl_3$ were added to the petri dish and left for 10 min to dissolve unreacted monomer and DMF to calculate the conversion. The $CDCl_3$ was transferred to the NMR tube. The gel was washed with chloroform (3x20 mL) and left for drying in a beaker capped with aluminum foil with holes overnight. Then it was dried in the desiccator overnight. The ^1H NMR spectra (before and after the reaction) revealed 23% conversion (DMF acted as the NMR standard).

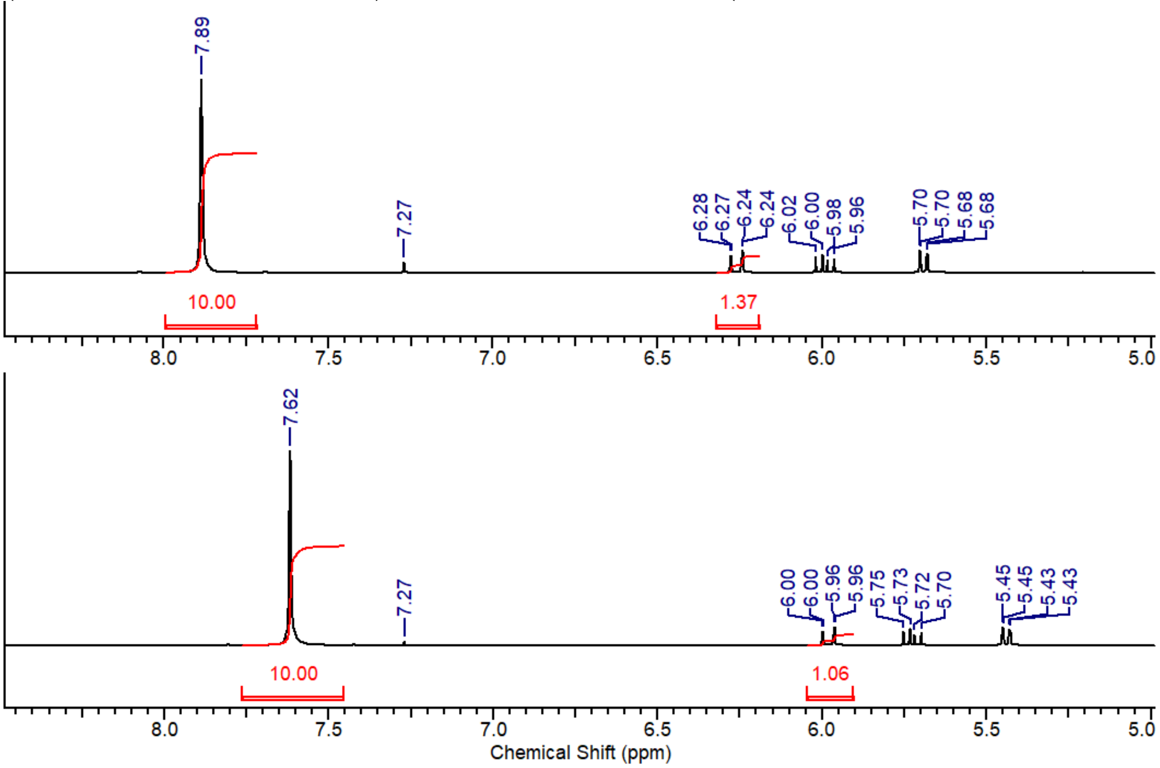

Figure S2. ^1H NMR spectra showing integrated values of the DMF standard (10.00) and the *n*-butyl acrylate monomer (1.37 to 1.06), indicating a 23% monomer conversion for the STEM-1-I25 gel. The top spectrum is from the initial reaction mixture and the bottom spectrum is from the reaction mixture after 4 hours.

General procedure for the swelling capacities analysis:

The given network material was taken and weighed in a 12 mL vial, and the given amount of the solvent was added to this vial, followed by the capping of the vial. It was left for swelling for 24 hours at room temperature. The left-over solvent was decanted, and the swollen network was transferred to a new vial. Then, the swollen material was weighed. The swelling capacity is calculated by the given formula, swelling capacity $(Q)=(W_s-W_d)/W_d$, where W_s and W_d are the weights of the swollen and dry networks, respectively.

Table S1. Swelling capacity analysis of networks.

Gel	Weight before swelling	Weight after swelling	Solvent	Swelling capacity (Q)
STEM-0-I10 gel	13 mg	111 mg	DMF (2.6 mL)	7.5
STEM-1-I10 gel	18 mg	188 mg	DMF (3.6 mL)	9.4
STEM-0-I25 gel	30 mg	307 mg	DMF (3.0 mL)	9.2
STEM-1-I25 gel	31 mg	226 mg	DMF (3.1 mL)	6.3

NMR Data

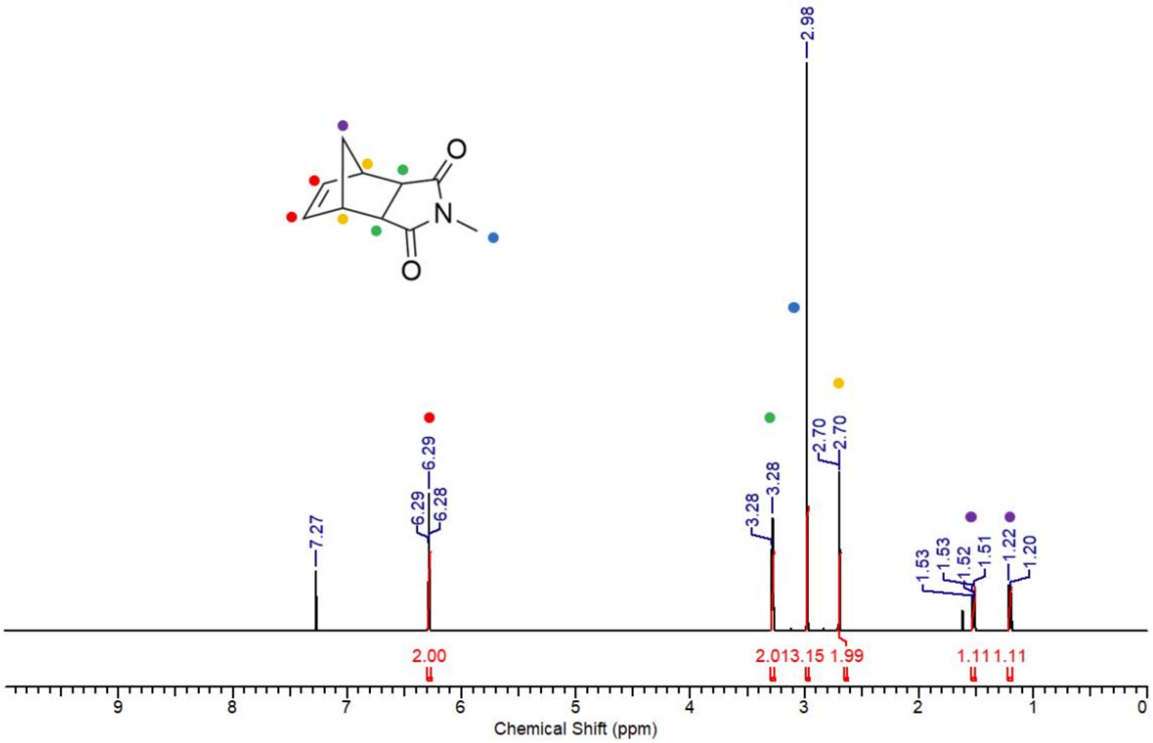

Figure S3. ^{1}H NMR spectrum (500 MHz, $CDCl_3$) of monomer.

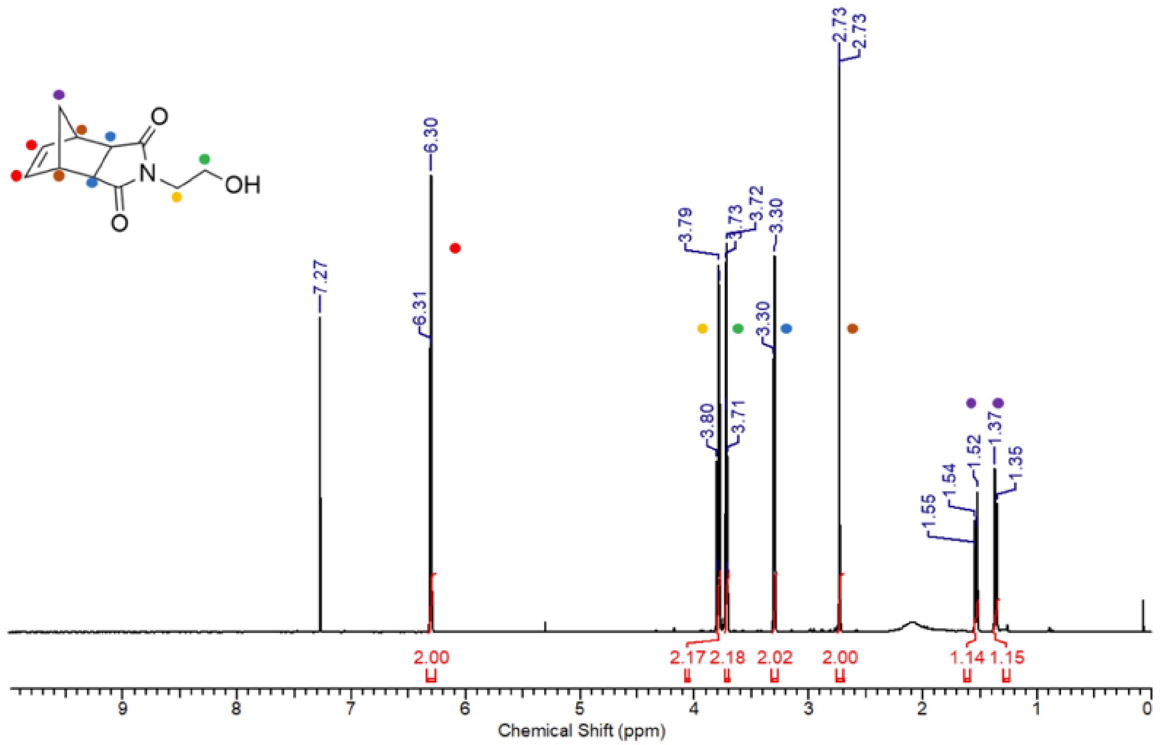

Figure S4. ^{1}H NMR spectrum (500 MHz, $CDCl_3$) of *exo*-*N*-hydroxyethyl-norbornenecarboximide.

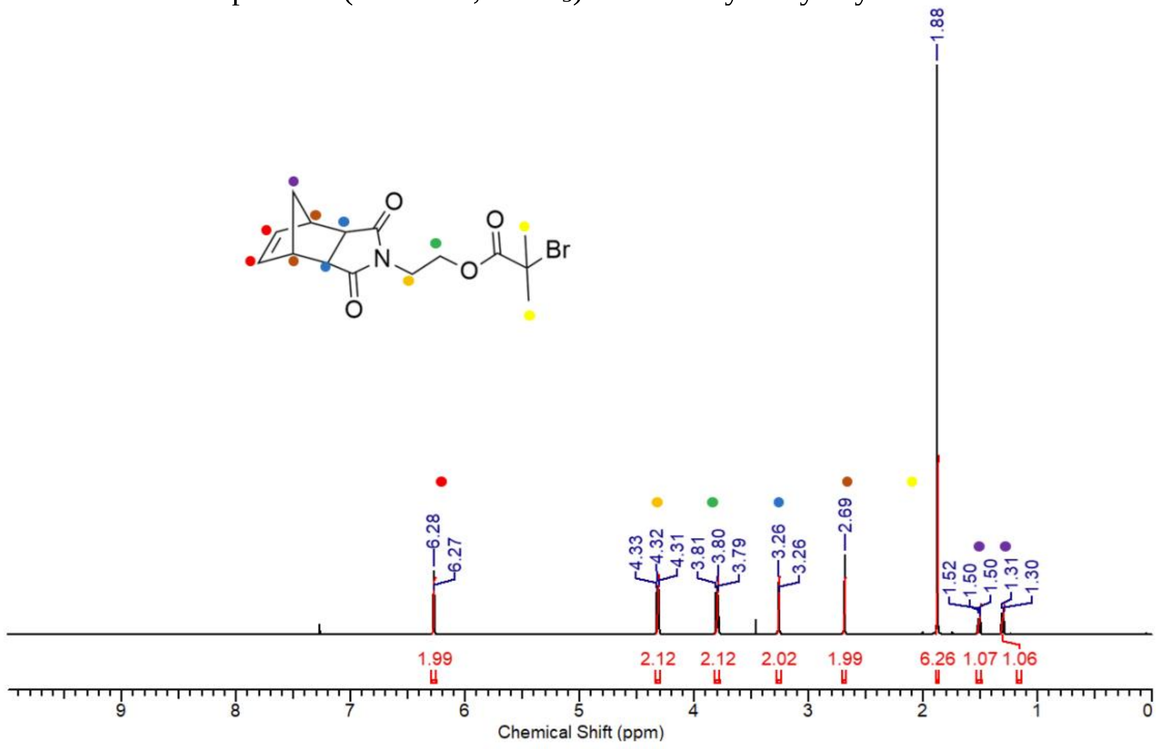

Figure S5. ^{1}H NMR spectrum (500 MHz, $CDCl_3$) of inimer.

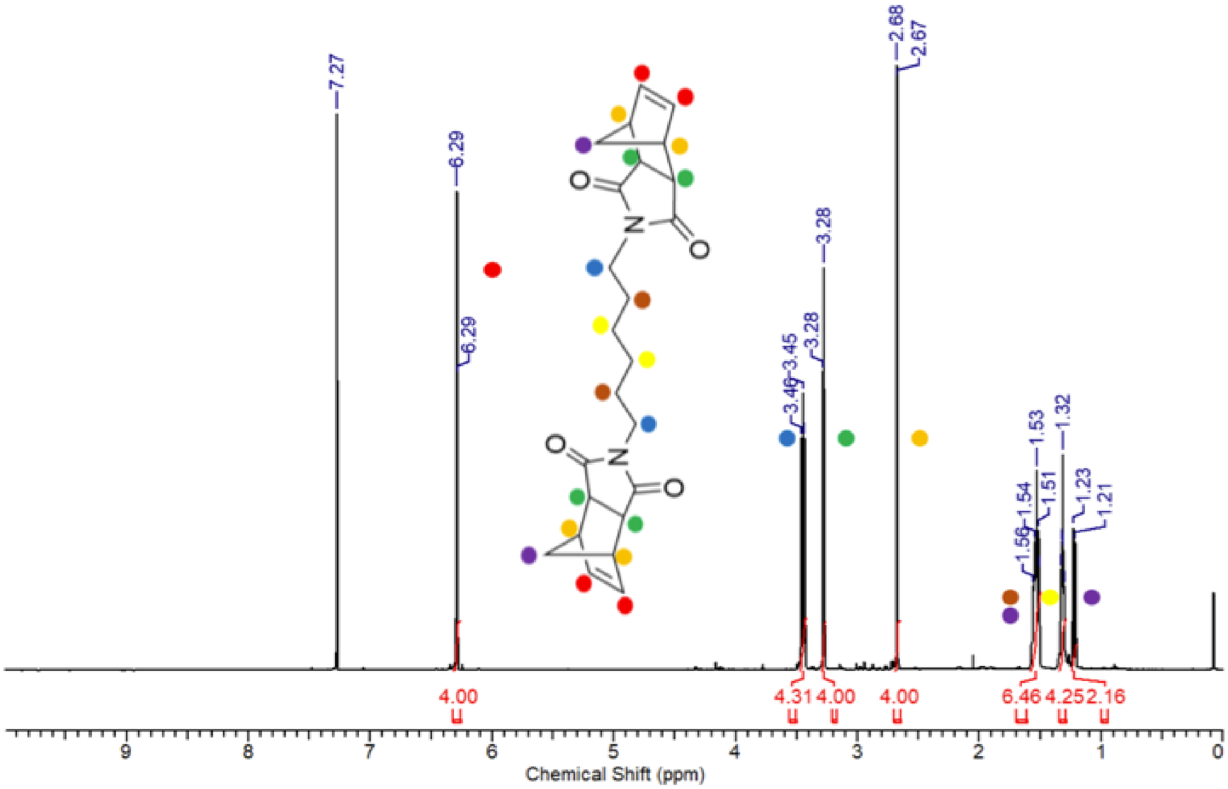

Figure S6. ^{1}H NMR spectrum (500 MHz, $CDCl_3$) of crosslinker.
